# Photophysical Properties of a Mn(I) Tricarbonyl PhotoCORM With 8‐Aminoquinoline Ligand: Insights From Theory

**DOI:** 10.1002/jcc.70338

**Published:** 2026-02-24

**Authors:** Daniele Belletto, Domenico Pisano, Ahmed M. Mansour, Gloria Mazzone, Tamer Shoeib, Emilia Sicilia

**Affiliations:** ^1^ Department of Chemistry and Chemical Technologies Università della Calabria Arcavacata di Rende Cosenza Italy; ^2^ Department of Chemistry, Faculty of Science Cairo University Cairo Egypt; ^3^ Department of Chemistry, School of Sciences and Engineering The American University in Cairo New Cairo Egypt

**Keywords:** CO‐releasing molecules, DFT, excited states, intersystem crossing, photoactivation, TDDFT

## Abstract

The photophysical properties of a recently synthesized photoCORM, the Mn(I) tricarbonyl complex fac‐[MnBr(CO)_3_(AQ)] (AQ = 8‐aminoquinoline), with promising cytotoxic activity against triple‐negative breast cancer, have been investigated by means of density functional theory (DFT) and time‐dependent DFT calculations. Simulations in various solvents (water, DMSO, THF) confirmed the Mn–CO bonds follow a Dewar–Chatt–Duncanson model, only slightly modulated by solvent polarity. The optimized computational method accurately reproduced the UV–vis spectrum, identifying the lowest energy excitations as metal‐to‐ligand charge‐transfer (MLCT) states with minor ligand‐to‐ligand charge‐transfer (LLCT) contributions, whose extent depends on solvent polarity. Exploration of the triplet excited‐state manifold revealed several low‐lying metal‐centered (^3^MC) states accessible from the initial singlet. High spin–orbit coupling and rapid intersystem crossing rates indicate that CO photorelease occurs through efficient population of these dissociative ^3^MC states, especially in polar media. These findings provide mechanistic insight into the photoactivation pathway of Mn(I)‐based photoCORMs and establish a robust computational framework for designing efficient CO‐releasing therapeutic agents.

## Graphical abstract

The computation of the photophysical properties of the Mn(I) *fac*‐[MnBr(CO)_3_(AQ)] photoCORM shows how different solvents can influence structural and light‐triggered behavior for CO molecules release, very likely occurring from triplet states with MC character.

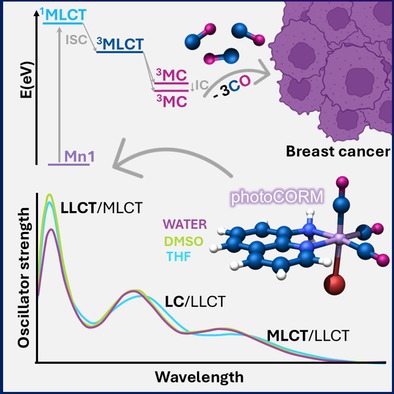

## 1 Introduction

Photoactivated Chemotherapy (PACT) agents are a class of compounds designed to be activated by light irradiation. Ideally, the chemical formula of nonirradiated PACT agents, having low toxicity and no biological activity in the dark, should change upon irradiation. Irradiated compounds become much more cytotoxic, allowing for a precise time‐ and spatially‐resolved control of their interaction with complex biological systems confining drug activity within diseased tissues with negligible side effects on normal tissues [1, 2, 3]. Even if any metal‐containing compound whose chemical formula changes due to the action of light and this change leads to some form of biological activity can be classified as a PACT agent. On the basis of the mechanism of action, inorganic PACT agents can be classified in four main categories [3]: (a) compounds that are reduced under light irradiation in the cellular environment causing the release of ligands and formation of the corresponding reduced active complexes that, together with the released ligands, can have both therapeutic effects; (b) compounds that, when irradiated, are susceptible to ligand substitution by solvent molecules, typically water. To both the solvated metal complex and released ligands can be ascribed the therapeutic effect; (c) metal complexes able to release, upon irradiation, small biological effector molecules such as NO and CO inactivated by the coordination to the metal center; (d) metallodrugs activated through the photomodification of a coordinated ligand. The attention here is focused on the subcategory of PACT agents belonging to the third class, which are metal complexes able to release CO molecules when irradiated.

Toxicity of CO, which being colorless, odorless, and tasteless is impossible to detect, is well known. CO in high concentration is harmful to humans due to its significantly higher affinity to hemoglobin than O_2_. Relevance of CO for therapeutic application, instead, has been acknowledged only recently [4, 5, 6]. CO is endogenously generated from heme due to the activity of heme oxygenase enzymes and is widely studied for its physiological actions including cytoprotective activity. Aiming at exploiting this protective action for therapeutic applications in medicine, CO‐releasing molecules (CORMs) have been synthesized and studied as prodrugs, in a secure and controlled manner, of potentially active doses of carbon monoxide to specific organs and tissues [7]. In particular, metal‐based photo‐CORMs, as they have been called by Ford et al. [8] have shown promising applications in photo‐chemotherapy of cancer [9, 10, 11]. To play their role of CO prodrugs, CORMs have to be readily soluble in water, sufficiently stable at room temperature, able to survive in the blood remaining active up to the desired targets and should produce only non‐toxic fragments after the CO release [12]. Typically, metals coming from the groups 7–9 of the periodic table (Mn, Fe, Co, Ru, Re) form stable photo‐CORMs and numerous investigations of such compounds can be found in the literature dealing with the details of the CO release process [13, 14, 15]. Among heavy metal‐containing CORMs, Mn‐based photo‐CORMs have attracted a great deal of attention as Mn is an essential trace bio‐element vital for all living organisms and its CO complexes ca be activated to release CO at low energy visible light. Mn(I)‐tricarbonyl photoCORMs have been the subject of intense studies due to their peculiar properties that fulfill many of the requirements for photochemotherapeutic applications [16, 17, 18, 19, 20].

In an earlier review, architectures, CO‐releasing activities, kinetics parameters, dark stability, quantification of the released CO molecules, and the nature of the solvents used in the previous studies were reported and tabulated [15]. On the basis of the information coming from both theoretical and experimental investigations [21, 22, 23, 24] the mechanism of labilization and release of CO molecules from metal carbonyl complexes is assumed to involve the transfer of electron density from the electron‐rich metal centers to π* MOs of the ligand frame via strong metal‐to‐ligand charge transfer (MLCT) transitions in the visible/near‐IR region causing a weakening of the metal‐C bond and, thereby, promoting the rapid CO photorelease. In metal carbonyl complexes, CO molecules bind to the metal center by both σ‐donation of electron density from the ligand to an empty metal d‐orbital and π‐back donation of metal d electrons to the antibonding π‐orbital of CO ligands. The CO release process triggered by light irradiation should be performed at ideal wavelengths falling in the so‐called therapeutic window (600–850 nm) where the penetration depth of light into tissues is the smallest for UV, medium for visible and reaches its maximum in the near IR region. The ancillary ligands play a decisive role in tuning, besides the rate of CO loss and the toxicity of the photoproducts, the MLCT transitions and the irradiation wavelength. Extended conjugation in the ancillary ligands together with the nature of the donor groups causes a stabilization of the lowest unoccupied MOs (LUMOs), while donor atoms directly bound to the metal center, such as chlorido and bromido anions, modulate the energy of the highest occupied MOs [21, 25].

**SCHEME 1:**
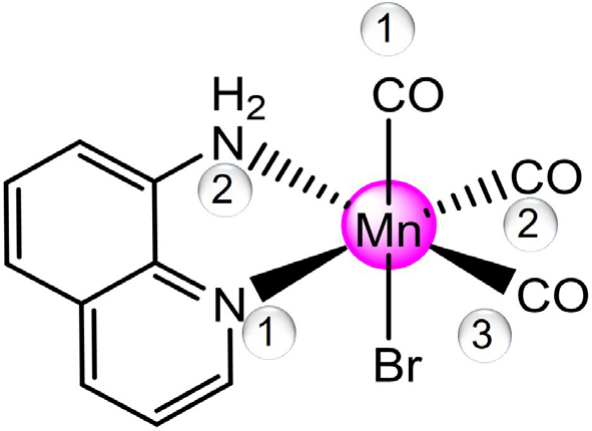
Schematic representation of the structure of the *fac*‐[MnBr(CO)_3_(AQ)] (AQ = 8‐aminoquinoline) Mn1 complex under investigation.

In the effort to synthesize Mn(I) CORMs able to fulfill all the requirements of photoCORMs, Mansour and coworkers have synthesized a tricarbonyl *fac*‐Mn(I) complex, *fac*‐[MnBr(CO)_3_(AQ)] (AQ = 8‐aminoquinoline) [26] shown in Scheme 1 and here named Mn1. The cytotoxicity of this complex has been tested toward triple negative breast cancer. Compared to all other published molecules, as ranked in the previously published review, the title complex has some desirable properties such as excellent solubility in a physiological media, a low‐power visible light requirement for CO release, and a long half‐life of CO release kinetics [15].

Motivated by the experimentally reported interesting cytotoxicity capabilities of such novel synthesized photoCORM, the photophysical properties of the complex Mn1 in different solvents: water, dimethylsulfoxide (DMSO) and tetrahydrofuran (THF) have been fully explored by means of density functional theory (DFT) and its time‐dependent extension (TDDFT). A complete structural characterization of the ground and excited states potentially involved in the photorelease of CO ligands was carried out to give further insight into the proclivity of the complex to release the CO ligands upon irradiation. The outcomes of the present investigation can be considered a good starting point for a subsequent detailed study of the mechanistic aspect of the whole CO release process occurring under irradiation.

## 2 Computational Details

All electronic structure calculations have been carried out with the Gaussian 16 package [27]. The ground‐state geometries of all species have been optimized at the DFT level using the B3LYP [28, 29] hybrid functional with Grimme's empirical dispersion correction (GD3) [30], and employing the 6‐31G(d,p) basis set to describe all atoms, including Mn and Br. For all geometry optimizations, water (*ε* = 78.4), DMSO (*ε* = 46.8) and THF (*ε* = 7.4) solvent effects have been taken into consideration via the Tomasi implicit polarizable continuum model (PCM) [31, 32], as implemented in Gaussian 16. Vertical electronic excitations and UV–vis absorption spectra have been obtained using the time‐dependent DFT in the Tamm–Dancoff approximation [33] (TDA‐DFT) on the optimized ground state geometries, applying the same functional and basis set reported above. The computational protocol has been defined after a preliminary benchmark considering a wide range of exchange‐correlation functionals: B3LYP, B3LYP‐D3, B3PW91 [28, 34], B97D [35], CAM‐B3LYP [36], M05 [37], M05‐D3, M06 [38], M06L [39], M11 [40], M052X [41], MN12L [42], MN15L [43], PBE [34, 44], PBE0 [45], TPSS [46], ωB97XD [47], coupled with 6‐31G(d,p) basis set for C, N, O, and Br atoms, while Mn has been described with LANL2DZ pseudo potential [48, 49, 50] and LANL2DZ basis set recontracted for the valence shell [51]. Final absorption spectra at TDA‐B3LYP‐D3/6‐31G(d,p) have been obtained by including 50 excitations in the calculation. Excited‐state potential energy surfaces have been further explored by optimizing the structures of the relevant excited singlet and triplet states, potentially involved in the complex photoactivation, in the condensed phase at the TDA‐DFT level. For triplet states, unrestricted Kohn‐Sham formalism [52, 53] has been applied on starting TDA‐DFT geometries. To analyze the character of the electronic transitions of the reference complex, a fragment based excited state analysis has been performed with the TheoDORE (theoretical density, orbital relaxation and exciton analysis) software package [54]. To establish the probability of triplet states population, spin–orbit coupling (SOC) matrix elements between the first excited singlet, S1, and the lowest lying triplet states have been computed with the TDA‐DFT approach, as implemented in the quantum chemical ORCA software [55, 56]. Scalar relativistic effects have been included through the zeroth order regular approximation (ZORA). The ZORA‐DEF2‐TZVP basis for all atoms, together with the SARC/J auxiliary basis within the RIJCOSX approximation, have been adopted to accelerate the Coulomb and exchange integral evaluation, following the ORCA manual recommendations. Stationary points along the reaction coordinates have been identified as minima or transition states based on the number of imaginary frequencies, 0 or 1, respectively. Zero‐point energy (ZPE) corrections have been included and to confirm the proper connection between transition states and their corresponding minima, intrinsic reaction coordinate (IRC) analyses [57, 58] have been carried out.

## 3 Results and Discussion

### 3.1 Structural and Electronic Properties

The main features of the investigated complex Mn1 have been reproduced in three solvents with high, medium and low polarity, that are, water, which mimics as best as possible the physiological environment, DMSO and THF [26]. To make explicit the different conditions, the complex is named as Mn1_W_, Mn1_D_, and Mn1_T_ if the simulation environment is water, DMSO, and THF, respectively.

A preliminary exploration on Mn1_W_ has been devoted to select the computational protocol that better reproduces the experimental findings, which are structural parameters, coming from X‐ray characterization, and absorption spectrum [26]. Outcomes are collected in Tables S1 and S2. It is worth remarking that not only the exchange and correlation functional plays a key role in reproducing electronic properties, but also the description of the metal center with or without a pseudopotential can have a significant impact, especially in obtaining the coordination bonds as similar as possible to the crystallographic structure. On the basis of the outcomes of this preliminary analysis, the most suited protocol is the combination of the B3LYP functional with the 6‐31G(d,p) basis set for all the atoms.

**SCHEME 2:**
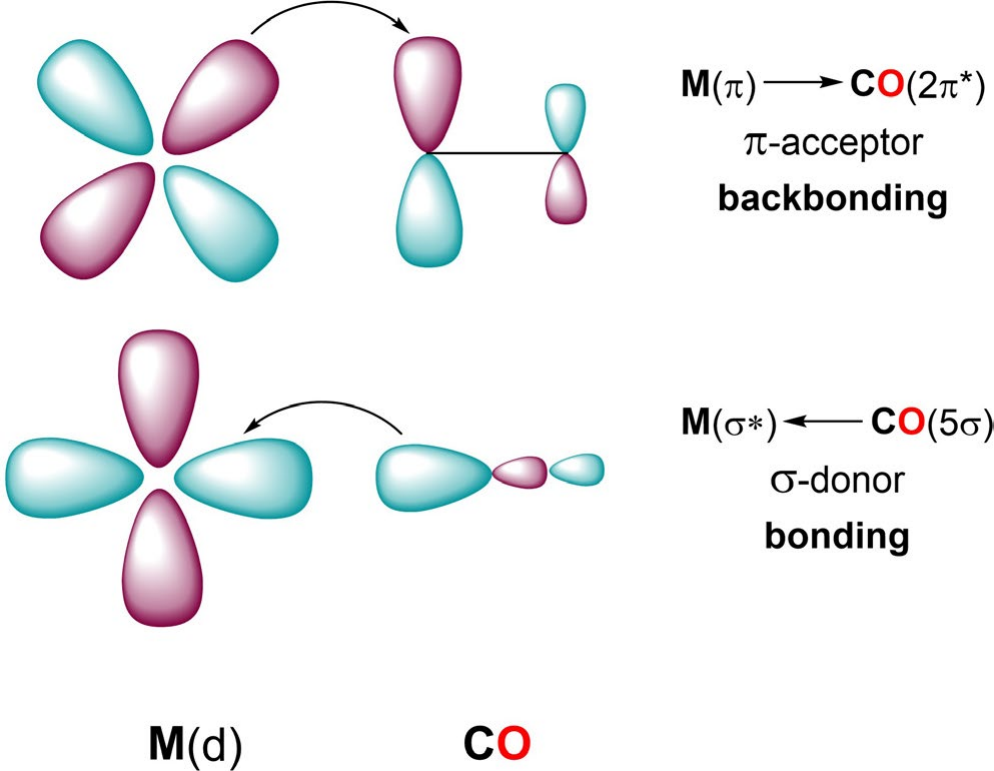
Schematic representation of the Dewar–Chatt–Duncanson (DCD) model of σ‐donation/π‐back donation in M–CO bonds.

The six‐coordinate optimized structure of the *fac*‐[MnBr(CO)_3_(AQ)] complex in the considered environments shows that the CO ligands, in a *fac* arrangement, occupy one axial and two equatorial positions. Coordination of carbon monoxide to metal centers occurs via two key interactions that, according to the Dewar–Chatt–Duncanson (DCD) model [59], are σ‐donation and π‐backdonation. σ‐donation involves electron density donation from a filled C‐centered orbital to an empty metal orbital, while electron density is transferred from a filled *t*_2*g*_ orbital of the metal to the low‐lying π* antibonding orbital of CO in π‐backdonation, as sketched in Scheme 2. The effects of backdonation are: (i) M–C bond strengthening due to the extra electron sharing, (ii) C–O bond weakening due to the transfer of electrons into an antibonding orbital and, as a consequence, (iii) lowering of the CO stretching frequency compared to the free CO. Such analysis has been done on the most stable structure of the complex in the three environments, and the outcomes are reported in Table 1.

**TABLE 1:** Selected structural parameters, bond distances in Å, and vibrational frequencies (cm^−1^) for the Mn1 complex in the three different environments and free CO ligand in vacuum.

	Mn1_W_	Mn1_D_	Mn1_T_	Free CO
Bond distances (Å)				
Mn–C1	1.786	1.786	1.785	
Mn–C2	1.804	1.804	1.804	
Mn–C3	1.796	1.796	1.797	
C1–O	1.160	1.160	1.160	1.138
C2–O	1.157	1.157	1.156	
C3–O	1.158	1.158	1.158	
Frequencies (cm^−1^)^a^				
ν_1_CO	1913	1914	1920	2122
ν_2_CO	1924	1925	1935	
ν_3_CO	2017	2017	2020	

*Note:* Numbering of C atoms is reported in Scheme 1.

^a^ Frequencies scaled (a scaling factor of 0.9608 is used for B3LYP/6‐31G(d,p) calculations).

As can be inferred from these data, the coordination of CO ligands to the metal center reflects a typical DCD interaction [59] as an elongation of the C–O bond and a consequent reduction of the CO stretching frequency is observed. The reported results also show that the coordination sphere of the metal does not undergo any significant change in the different environments as all the bond distances between the metal and CO ligands remain essentially the same. The simulated IR spectrum in water shows three bands at 1913, 1924, and 2017 cm^−1^ assigned to the symmetric and antisymmetric CO stretches of the Mn(CO)_3_ portion of the complex. A quite superimposable situation is found for the complex optimized in the less polar solvents, especially in DMSO. The simulated stretching wavenumbers are in good agreement with the experimental values (1896, 1932, and 2021 cm^−1^), corresponding to the symmetric and two antisymmetric stretching vibrational modes, respectively. The equatorial Mn–C bonds, in all environments, are longer than the axial one as a consequence of the *trans* effect of the nitrogen atoms of the AQ ligand that weaken the bonds with the ligands in *trans* position. Additionally, the aromatic nitrogen has a stronger effect than the aliphatic one, causing the Mn‐C2 bond to be the longest one and an opposite effect on the C–O bond length.

Comparison with the X‐ray structural data reveals a slight overestimation of the coordination bond lengths, ranging from 0.001 to 0.042 Å, except for the Mn–C1 and Mn–C3 distances, which are underestimated by approximately 0.02 Å. The deviations in the valence angles fall between −2.6° and 3.9°, independent of the environment used for geometry optimization. It is worth noting that the simulated parameters were obtained using an implicit solvent model, whereas the experimental values refer to the crystal structure; therefore, the observed discrepancies lie within the range of typical errors reported in similar studies.

To estimate the amount of π‐backdonation, the natural bond order (NBO) analysis was carried out and the second‐order perturbation energy term, *E* ^(2)^, which represents the interaction energy between donor (i) and acceptor (j) orbitals, has been analyzed. It is calculated using the energy difference between the acceptor and donor orbitals (*ε*_*j*_ *− ε*_*i*_) and the corresponding Fock matrix element obtained from NBO analysis. To evaluate the extent of backdonation, the predominant π‐type back‐bonding interactions toward each CO ligand (namely CO1, CO2, and CO3 as labeled in Scheme 1) have been summarized in Table 2. Such analysis evidence, again, a very similar behavior of the complex in the different environments, especially in water and DMSO. π‐backdonation from a filled metal orbital to the antibonding π* of each CO is slightly attenuated in THF but remains worthy of note. If compared with the behavior explored very recently of the reference complex [Mn(CO)_6_]^+^, the amount of backdonation in Mn1 is considerably higher, up to one order of magnitude [25]. With the aim to evaluate more in detail the influence on π‐backdonation of the other ligands, namely AQ and Br, the same analysis has been carried out on the reference complex and the related complexes where CO ligands have been replaced alternatively by the AQ ligand, obtaining the [Mn(CO)_4_(AQ)]^+^ complex, and Br ligands, thus obtaining the [MnBr(CO)_5_] complex (Table S3). The NBO analysis of these complexes shows that the most significant influence on the synergistic σ‐donation/π‐backdonation that is observed for all the CO ligands comes from the AQ ligand in equatorial positions and Br for the axial one. Indeed, the equatorial CO ligands in [Mn(CO)_4_(AQ)]^+^ are characterized by the largest back‐donation (more than 90 kcal mol^−1^), while in the case of the [MnBr(CO)_5_] complex the axial CO ligand is the only one with a backdonation around 88 kcal mol^−1^ and remains in the range 24–30 kcal mol^−1^ for all the other CO ligands. Backdonation does not exceed 13 kcal mol^−1^ in [Mn(CO)_6_]^+^.

**TABLE 2:** Computed NBO analysis for the investigated complexes. *E* ^(2)^ energies are in kcal mol^−1^, while ***ε***_***j***_ ***— ε***_***i***_ and ***F***_**(*i*,*j*)**_ are in arbitrary units.

Complex	Donor (*i*)	Acceptor (*j*)	*E* ^(2)^	*E* ^(2)^_TOT_ ^a^	*ε*_*j*_ *– ε*_*i*_	*F*_(*i*,*j*)_
Mn1_w_	n_(1)_Mn	π*_(1)_CO1	10.59	102.33	0.20	0.045
	n_(3)_Mn	π*_(1)_CO1	91.74		0.05	0.074
	n_(2)_Mn	π*_(1)_CO2	6.15	83.17	0.19	0.034
	n_(3)_Mn	π*_(1)_CO2	77.02		0.06	0.072
	n_(1)_Mn	π*_(1)_CO3	1.88	89.69	0.19	0.018
	n_(2)_Mn	π*_(1)_CO3	14.62		0.18	0.05
	n_(3)_Mn	π*_(1)_CO3	73.19		0.04	0.06
Mn1_D_	n_(1)_Mn	π*_(1)_CO1	10.57	102.19	0.20	0.045
	n_(3)_Mn	π*_(1)_CO1	91.62		0.05	0.074
	n_(2)_Mn	π*_(1)_CO2	6.23	83.02	0.19	0.034
	n_(3)_Mn	π*_(1)_CO2	76.79		0.06	0.072
	n_(1)_Mn	π*_(1)_CO3	1.85	89.40	0.19	0.018
	n_(2)_Mn	π*_(1)_CO3	14.68		0.18	0.05
	n_(3)_Mn	π*_(1)_CO3	72.87		0.04	0.06
Mn1_T_	n_(1)_Mn	π*_(1)_CO1	10.48	98.87	0.20	0.045
	n_(3)_Mn	π*_(1)_CO1	88.39		0.06	0.074
	n_(2)_Mn	π*_(1)_CO2	6.79	78.84	0.19	0.036
	n_(3)_Mn	π*_(1)_CO2	72.05		0.06	0.07
	n_(1)_Mn	π*_(1)_CO3	2.59	84.05	0.19	0.022
	n_(2)_Mn	π*_(1)_CO3	14.83		0.18	0.05
	n_(3)_Mn	π*_(1)_CO3	66.63		0.05	0.058

^a^ Sum of π‐backdonation to the same acceptor from different metal lone pairs.

### 3.2 Electronic Spectrum

A detailed benchmark on the exchange and correlation functionals (Table S1) has been carried out to select the most suitable computational protocol able to reproduce as much as possible the electronic spectrum of Mn1, paying major attention to the maximum absorption wavelength *λ* ^max^, pivotal for tissue penetration. For this purpose, generalized gradient approximation (GGA) (PBE, B97D), meta‐GGA (TPSS, M06L, MN12L, MN15L), hybrid GGA (B3LYP, B3PW91, PBE0), hybrid meta‐GGA (M05, M06, M05‐2X, MN15), and long‐range corrected (wB97XD, cam‐B3LYP, M11) functionals have been tested in reproducing the absorption spectrum of the complex within the TDDFT formalism. It is worth mentioning that, differently from the recorded spectrum, for which the wavelength corresponding to the center of the shoulder has been provided, in most cases simulations return at least two close electronic transitions ascribable to the same charge‐transfer as responsible for the lowest‐energy band. Therefore, the average wavelength of the two electronic transitions was considered as the position of such a band.

From such exploration emerges that, except for some hybrid and long‐range corrected functionals that overestimate the experimental *λ* ^max^ by no more than 27 nm, the overestimation of *λ* ^max^ computed by the other types of functionals falls in a wide range of 30–130 nm with the GGA functionals, PBE and B97D, and M05‐2X that go beyond 140 nm and MN15 that completely fails in reproducing such quantity. Among the functionals found to be reliable, the B3LYP one, including dispersion corrections, has been selected as the most suited in reproducing both structural and absorption properties.

**FIGURE 1:**
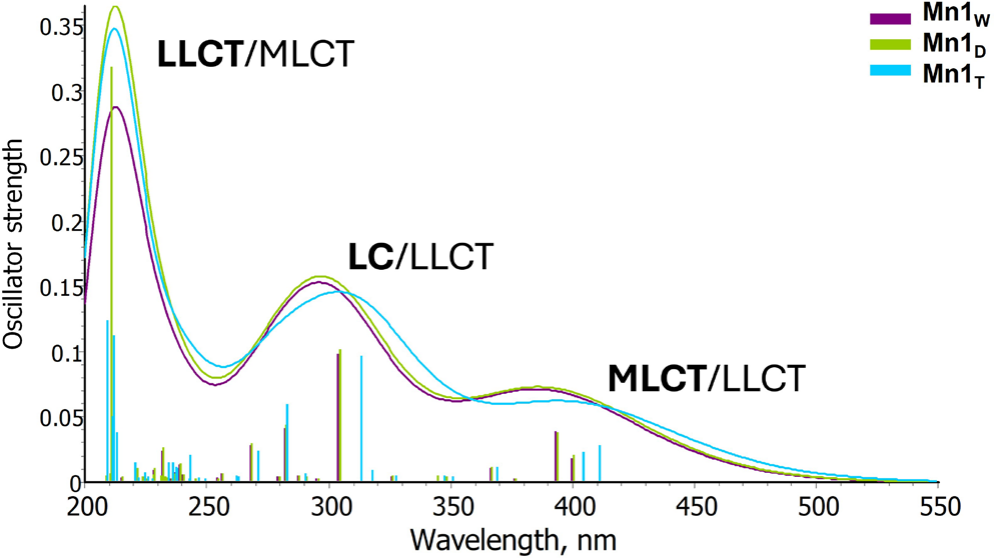
Computed absorption spectra of Mn1 optimized in water (grape line), DMSO (green line), and THF (cyan line), Mn1_W_, Mn1_D_, and Mn1_T_, respectively. The major contribution character (MLCT, LC, and LLCT) to each band is reported in bold.

**FIGURE 2:**
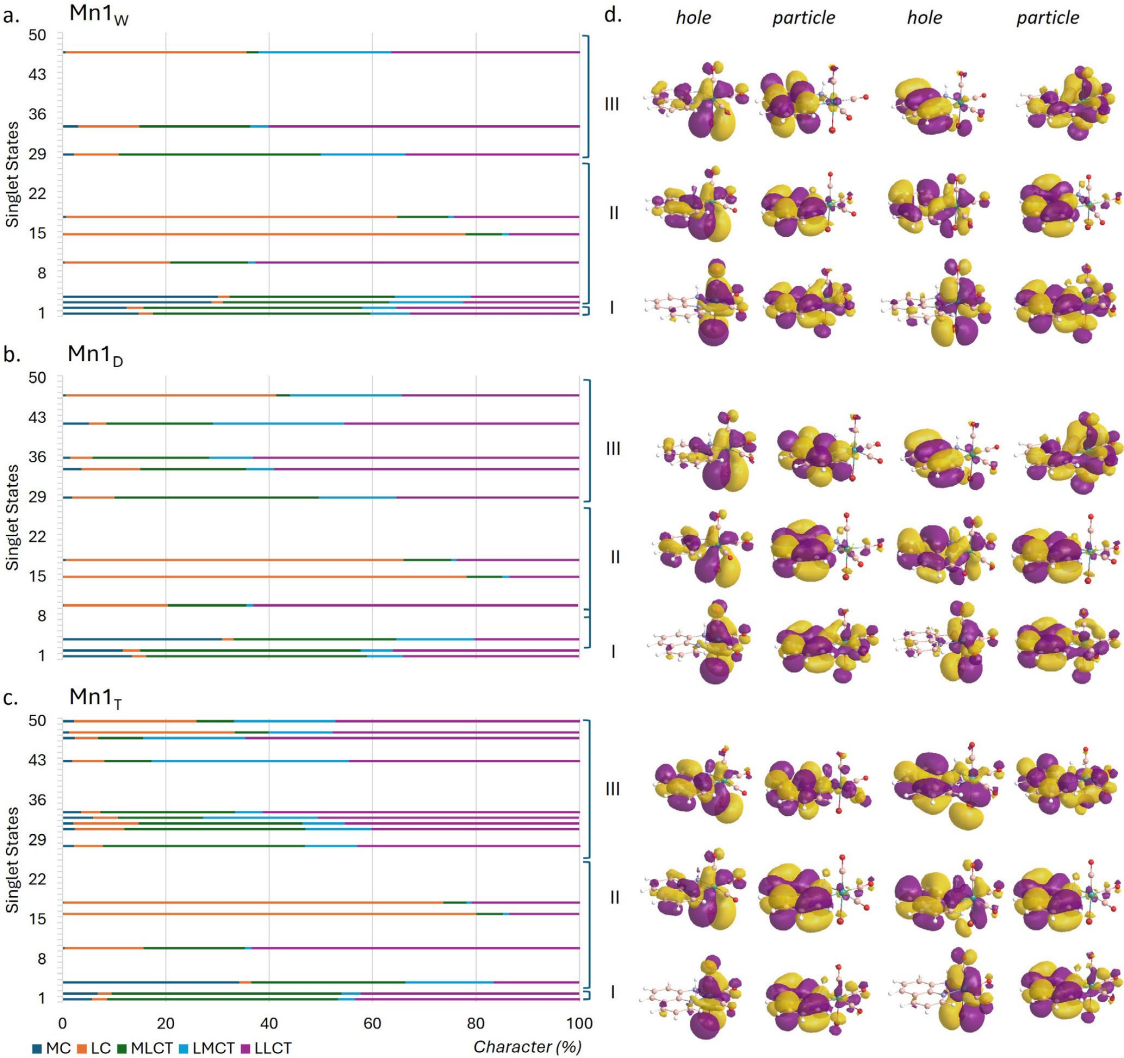
Decomposition analysis of excited singlet states of the complex in (a) water, (b) DMSO, and (c) THF and (d) NTOs (hole and particle) of the two most significant transitions for each band generated with Chemissian software v4.67 (isovalue 0.02 a.u.). Only transitions with oscillator strength greater than 0.01 have been considered.

The TDA‐B3LYP‐D3 calculated UV–vis spectrum of the Mn1 complex in the three environments is reported in Figure 1 and more detailed information is provided in Table S4. Figure 2, instead, reports the natural transition orbitals (NTOs) associated with the most significant electronic transitions, together with the fragment‐based analysis used for assigning state character. To assign the character to each band of the whole spectrum, only electronic transitions with oscillator strength greater than 0.01 have been taken into consideration.

The spectrum is characterized by three bands in the range 200–550 nm, though the band located at higher wavelengths can be considered a shoulder, as experimentally reported [26]. Such a band is quite similar for high and medium polar solvents, that are water and DMSO, while the absorption in this region is red shifted by 12 nm with respect to water environment in THF, somewhat mirroring what found experimentally. The two electronic transitions associated with this band start from an orbital primarily located on the metal, HOMO (H) and H‐1, respectively, and end into the LUMO which is outspread on the whole π‐system (Figure 2). Therefore, the lowest energy band can be predominantly ascribed, in all cases, to MLCT, though looking at the decomposition analysis a minor contribution of LLCT can be spotted. The latter becomes more pronounced in THF, being by more than 40% similar to the MLCT contribution (45% for both transitions). Going up in energy, a second band is found in the 250–350 nm region and picked at around 290 nm. In all the considered environments, this band is originated by few transitions that involve inner orbitals, like H‐3 and H‐5, while the LUMO is again the terminal orbital. The decomposition analysis returns a mixed character for this band, LC/LLCT, for a CT involving mainly the AQ ligand. Analogously to the first band, the THF lower polarity red shifts the electronic transitions also in this region, even if the involved orbitals remain the same. The last band, between 200 and 250 nm, is essentially of LLCT type, originating by charge transfer from the CO ligands to the AQ one and *vice versa*, though a minor contribution to the CT from the metal to the AQ ligand gives a mixed character LLCT/MLCT. As for the other bands, water and DMSO display very similar behavior as in both environments the electronic transitions that mainly contribute to this band are H‐5 → L + 1 and H‐6 → L + 2. On the contrary, other orbitals are involved (H‐3 → L + 5 and H‐7 → L + 1, H‐5 → L + 4) when THF is considered.

### 3.3 Excited States Description

**FIGURE 3:**
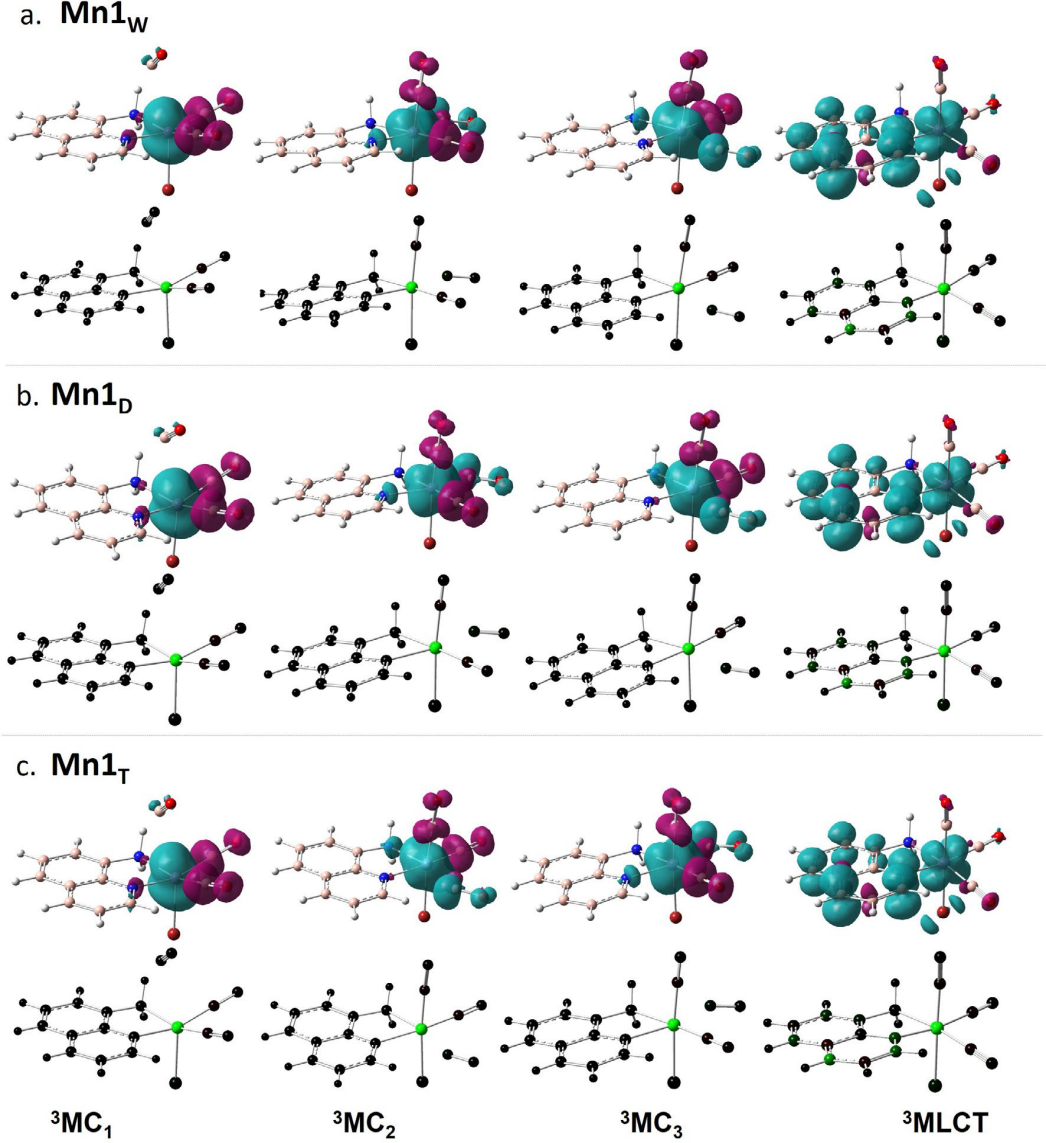
Spin density distribution of the excited triplet states located in (a) water, (b) DMSO, and (c) T environments. All the isosurfaces have been plotted with an isovalue of 0.004 a.u. Mulliken spin population for each state is also reported in symmetric color scale, for which green means nonzero spin density where unpaired electrons are localized.

The generally accepted mechanism for ligand photorelease begins with excitation to a higher‐lying singlet state. Due to the singlet state much shorter lifetime (10^−8^ vs 10^−3^ s for the triplet state), a rapid radiationless intersystem crossing (ISC) should occur from the singlet excited state to populate a triplet state prior to ligand release [60, 61, 62]. To locate and characterize all the potentially relevant triplet states, we have first optimized the 20 triplet excited states located within the time‐dependent approximation. The resulting guesses have been then used for performing unrestricted DFT calculations to obtain more reliable triplet states. In all the considered environments, the 20 triplet excited states converged into four states reported in Figure 3 ordered by energy increasing values as will be further discussed in the next section.

The main structural parameters for each state have been collected in Table 3, where the ground and first excited singlet state parameters are included as well for a direct comparison. First, despite the mixed nature of the electronic transition allowing the population of the first excited state (Figure 2), the optimization of such state at TDA‐B3LYP‐D3 level yields a state with MLCT character in all the environments. The population of the bright state is accompanied by marginal structural rearrangements that mainly regard the elongation of the Mn–C bonds and a decrease of the bond distances between the metal center and both Br and AQ ligands, confirming the MLCT character involving the π‐system of the AQ ligand. To characterize the triplet state, spin density distribution and Mulliken spin population have been used. The green color highlights that the unpaired electrons are exclusively located on the metal center as well as the spin density distribution mainly located on the metal center or in its close proximity.

**TABLE 3:** Key bond length (Å) for all the characterized excited states and relative adiabatic energy (Δ*E*, eV) calculated with respect to the ground state.

	Mn1_W_ 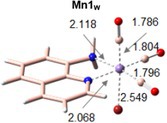					Mn1_D_ 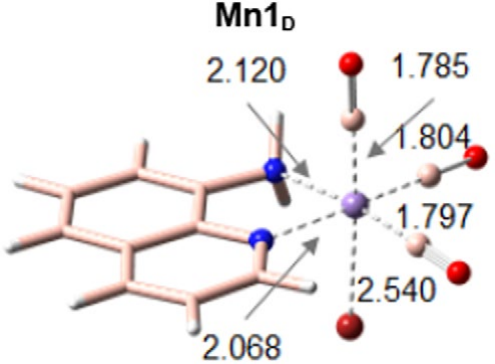					Mn1_T_ 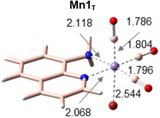				
	^3^MC_1_	^3^MC_2_	^3^MC_3_	^3^MLCT	S1	^3^MC_1_	^3^MC_2_	^3^MC_3_	^3^MLCT	S1	^3^MC_1_	^3^MC_2_	^3^MC_3_	^3^MLCT	S1
MnC1	3.001	1.860	1.857	1.876	1.864	3.010	1.856	1.860	1.876	1.863	2.995	1.876	1.853	1.873	1.861
MnC2	1.825	1.811	2.199	1.918	1.841	1.824	2.199	1.811	1.918	1.840	1.824	1.815	2.185	1.914	1.837
MnC3	1.814	2.208	1.804	1.826	1.855	1.814	1.804	2.206	1.826	1.855	1.813	2.193	1.808	1.826	1.854
MnBr	2.504	2.499	2.486	2.458	2.409	2.502	2.485	2.498	2.458	2.408	2.490	2.490	2.477	2.452	2.402
MnN1	2.064	2.110	2.200	1.961	2.040	2.064	2.202	2.111	1.961	2.040	2.066	2.115	2.208	1.961	2.046
MnN2	2.120	2.260	2.178	2.112	2.090	2.120	2.179	2.261	2.112	2.090	2.124	2.279	2.186	2.114	2.092
Δ*E*	1.45	1.59	1.59	2.22	2.78	1.45	1.59	1.59	2.22	2.77	1.47	1.58	1.58	2.20	2.67

Therefore, the lowest‐lying three triplet states have all metal‐centered (MC) character, and are, as a consequence, dissociative states facilitating the release of the CO ligands. They are all arranged in a quite distorted geometry with respect to the reference octahedral‐like structure of the ground state. The main difference relies on the CO ligand detaching from the metal: in ^3^MC_1_ the Mn–C coordination bond elongated up to 3.001 Å is that formed with the axial CO ligand, as it clearly appears in Figure 3. The second and third states, instead, are characterized by the detachment of one of the equatorial CO ligands, and are labeled as ^3^MC_2_ and ^3^MC_3_, that appear equally stable in all the considered environments.

The last located triplet excited state exhibits a structure quite similar to that of the singlet excited one, characterized by the slight elongation of the Mn–C distances and shortening of the Mn–N ones. The unpaired electrons are, indeed, shared between the metal and part of the aromatic moiety of the AQ ligand.

### 3.4 ISC Rates

**FIGURE 4:**
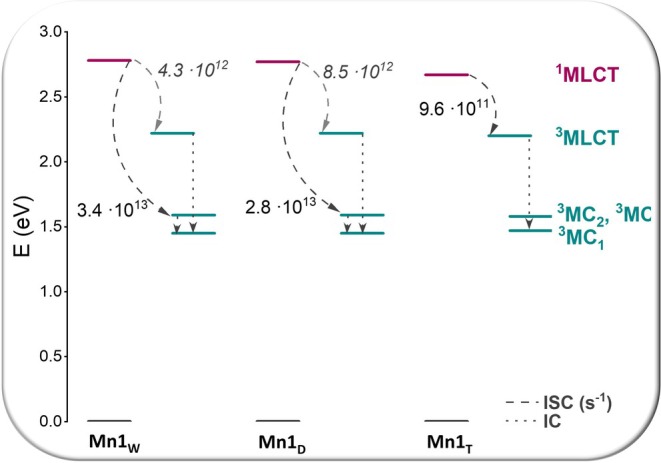
Mn1 energy level diagram and the most probable deactivation mechanisms of the ^1^MLCT bright state in the three considered environments.

Since direct population of triplet excited states is forbidden by selection rules, their population typically occurs through a highly probable radiationless ISC, which in metal complexes is very often driven by SOC. SOC is a relativistic phenomenon that influences both angular momentum and spin, causing a mixing of orbital and spin degrees of freedom and thereby enabling the coupling of electronic states with different multiplicities. According to Fermi's Golden Rule, estimating the kinetics of ISC and calculating the corresponding rate constant (*k*_ISC_) requires: (i) the relative energies of the states involved in the ISC process at their equilibrium geometries, (ii) a comprehensive description of all vibrational wavefunctions to evaluate the vibrational coupling between different electronic states, and (iii) the estimation of SOC matrix elements. The energy splitting between the coupled states has been determined as described in the previous section. Vibrational modes and the corresponding Hessian matrices for each excited state were obtained from the optimized geometries used to compute the adiabatic energy splitting. The SOC values between the relevant electronic states have been evaluated as the expectation values of the mean‐field spin–orbit operator. The key computed quantities, energy splittings, SOC values, and *k*_ISC_, are summarized in Table 4, and the most relevant results are illustrated in Figure 4, which depicts the most probable behavior in the three examined environments.

**TABLE 4:** Adiabatic energy splitting (ΔE), spin‐orbit coupling values (SOC) and ISC rates *k*_ISC_ (s^−1^) computed for ^1^MLCT deactivation in different environments.

		^1^MLCT–^3^MC_1_	^1^MLCT–^3^MC_2_	^1^MLCT–^3^MC_3_	^1^MLCT–^3^MLCT
Mn1_W_	Δ*E*	10,747	9603	9575	4503
	SOC	4.48	123.03	197.39	51.07
	*k*_ISC_	2.11 × 10^3^	4.29 × 10^9^	3.42 × 10^13^	4.26 × 10^12^
Mn1_D_	Δ*E*	10,668	9539	9511	4437
	SOC	4.43	123.50	197.78	49.62
	*k*_ISC_	3.91 × 10^3^	5.74 × 10^9^	2.83 × 10^13^	8.50 × 10^12^
Mn1_T_	Δ*E*	9721	8799	8776	3767
	SOC	5.16	132.12	204.57	94.45
	*k*_ISC_	2.36 × 10^4^	1.21 × 10^3^	2.81 × 10^8^	9.59 × 10^11^

*Note:* Energy splitting and spin–orbit coupling are in cm^−1^.

According to the data in Table 3, the complex can, in principle, access four deactivation pathways of the bright state via ISC in all examined environments, leading to population of the triplet states described above. Among these, the three lowest triplet states possess MC character, while the less stable one exhibits MLCT character.

The relative energies reported in Table 3 show that the MLCT triplet state lies very close in energy to the corresponding singlet state with which it can couple, suggesting that this could be the state most likely involved in the ISC process.

In contrast to the bond analysis discussed above, which indicates that different environments do not affect the most stable configuration of the Mn1 complex, the behavior associated with the spin–flip process leading to triplet state population shows notable differences. The most likely deactivation pathway of the bright state appears to be similar in water and DMSO but differs significantly in THF. In the latter case, as expected, the most probable coupling occurs between ^1^MLCT and ^3^MLCT states, with a *k*_ISC_ of 9.6 × 10^11^ s^−1^. The rate constants associated with such channel in the other solvents, instead, even if larger than in THF, are one order of magnitude smaller than the ISC process involving the ^3^MC_3_ state, the rate constant being 3.4 × 10^13^ s^−1^ in water. Even though all the most favored processes are accompanied by considerable SOC values, in THF the energy splitting plays a pivotal role as well. As extensively reported in the literature [60, 61, 62], the MC state is the most likely to be involved in ligand photorelease. Therefore, one of the three identified MC states could play a role in the CO release process.

## 4 Conclusion

Comprehensive DFT and TDDFT analyses of the *fac*‐[MnBr(CO)_3_(AQ)] complex have clarified the electronic factors governing its photoinduced CO‐release activity. Structural and spectroscopic data demonstrate that the coordination environment and π‐backdonation pattern remain largely invariant across solvents of different polarity, while the electronic excitation energies are subtly modulated. The lowest lying singlet excited state exhibits a predominant MLCT character, serving as the primary photoactive channel. Subsequent ISC efficiently populates triplet MC states responsible for CO labilization. Kinetic analysis reveals that the ISC rate constants are markedly enhanced in polar solvents, suggesting that aqueous environments favor photoactivation, consistent with its biological relevance. These results elucidate the interplay between solvent effects, charge‐transfer dynamics, and SOC in dictating CO‐release efficiency. The present computational work represents a necessary foundation for further in‐depth investigations of the mechanistic aspects of the process, which would enable the identification of key factors that could be exploited to improve the performance of this class of complexes.

## Author Contributions

The manuscript was written through the contributions of all authors. All authors have given approval to the final version of the manuscript.

## Funding

This research was supported by the Ministry of Foreign Affairs and International Cooperation of Italy and Science, Technology & Innovation Funding Authority of Egypt under grant number 47576 for the project “Novel Photo‐activated Carbon Monoxide Releasing Materials for Biomedical Applications” PGR02103.

## Conflicts of Interest

The authors declare no conflicts of interest.

## Supporting information


**Table S1:** Benchmark for selecting the computational protocol.
**Table S2:** Comparison between crystallographic and optimized structure of Mn(I) at B3LYP‐D3/6‐31G(d,p) level in the three environments considered.
**Table S3:** Computed NBO analysis for the reference complexes [Mn(CO)_6_]^+^, [Mn(CO)_4_(AQ)]^+^, MnBr(CO)_5_.
**Table S4:** TDDFT outcomes of Mn1 complex in water, DMSO and THF environments.

## Data Availability

The data that support the findings of this study are provided in the Supporting Information. Additional data are available from the corresponding author upon reasonable request.
