# Supplementary material for: Photophysical Properties of a Mn(I) Tricarbonyl PhotoCORM With 8‐Aminoquinoline Ligand: Insights From Theory
Source: J Comput Chem. 2026 Feb 24;47(6):e70338. doi: 10.1002/jcc.70338 (PMC12932087; doi:10.1002/jcc.70338)
Supplement: Supplementary file 1 — Table S1: Benchmark for selecting the computational protocol. Table S2: Comparison between crystallographic and optimized structure of Mn(I) at B3LYP‐D3/6‐31G(d,p) level in the three environments considered. Table S3: Computed NBO analysis for the reference complexes [Mn(CO)6]+, [Mn(CO)4(AQ)]+, MnBr(CO)5. Table S4: TDDFT outcomes of Mn1 complex in water, DMSO and THF environments. [file JCC-47-0-s001.docx]

**Supporting Information**

Photophysical Properties of a Mn(I) tricarbonyl PhotoCORM with 8-aminoquinoline ligand: Insights from theory

Daniele Belletto,^[a]^ Domenico Pisano,^[a]^ Ahmed M. Mansour,^[b,c]^ Gloria Mazzone,^[a]^ Tamer Shoeib,^[d]^ Emilia Sicilia^[a]^

1. Department of Chemistry and Chemical Technologies, University of Calabria, 87036 Rende, Italy;
2. Department of Chemistry, Faculty of Science, Cairo University, Gamma Street, Giza, 12613, Egypt;
3. Department of Chemistry, United Arab Emirates University, Al-Ain, United Arab Emirates.
4. Department of Chemistry, The American University in Cairo, New Cairo, 11835, Egypt.

Correspondence to: Gloria Mazzone (E-mail:*gloria.mazzone@unical.it)*

**Table of Contents**

- **Table S1:** Benchmark for selecting the computational protocol **S2**
- **Table S2:** Comparison between crystallographic and optimized structure of Mn(I) at B3LYP-D3/6-31G(d,p) level in the three environments considered **S3**
- **Table S3:** Computed NBO analysis for the reference complexes [Mn(CO)_6_]^+^, [Mn(CO)_4_(AQ)]^+^, MnBr(CO)_5_ **S4**
- **Table S4**: TDDFT outcomes of Mn1 complex in water, DMSO and THF **S5**
- **References** **S6**

**Table S1**

Benchmark for selecting the computational protocol

*TD-DFT UV-Vis spectra from different exchange and correlation functionals on the optimized structure at level of theory B3LYP-D3/6-31G(d,p) and LANL2DZ with its recontructed valence shell on Mn of Mn1_W_ complex.*

|  | Transition^a^ | λ (nm) | E (eV) | *f* |
| --- | --- | --- | --- | --- |
| *exp* ^b^ |  | *368* |  |  |
| B3LYP | 1  2 | 398  392 | 3.12  3.17 | 0.014  0.024 |
| B3LYP-D3 | 1  2 | 398  392 | 3.12  3.17 | 0.014  0.024 |
| B3PW91 | 1  2 | 399  392 | 3.11  3.17 | 0.017  0.025 |
| camB3LYP | 1  2 | 393  386 | 3.15  3.21 | 0.004  0.017 |
| B97D | 1  2 | 520  510 | 2.39  2.43 | 0.010  0.025 |
| ωB97XD | 1  2 | 390  383 | 3.18  3.24 | 0.004  0.017 |
| TPSS | 1  2 | 490  480 | 2.53  2.58 | 0.013  0.026 |
| PBE | 1  2 | 531  519 | 2.34  2.39 | 0.009  0.027 |
| PBE0 | 1  2 | 401  394 | 3.09  3.14 | 0.008  0.021 |
| M05 | 1  2 | 486  480 | 2.55  2.58 | 0.004  0.010 |
| M05-D3 | 1  2 | 486  480 | 2.54  2.58 | 0.004  0.010 |
| M06 | 1  2 | 471  463 | 2.63  2.68 | 0.004  0.012 |
| M06L | 1  2 | 472  465 | 2.63  2.67 | 0.013  0.031 |
| M052X | 1 | 533 | 2.33 | 0.007 |
| M11 | 1 | 494 | 2.51 | 0.006 |
| MN12L | 1  2 | 401  396 | 3.09  3.13 | 0.002  0.032 |
| MN15 | 1 | 795 | 1.56 | 0.003 |
| MN15L | 1  2 | 433  426 | 2.87  2.91 | 0.018  0.039 |
| B3LYP-D3^c^ | 1  2 | 398  391 | 3.12  3.17 | 0.014  0.031 |
| B3LYP-D3^d^ | 1  2 | 400  393 | 3.10  3.16 | 0.018  0.038 |
| a. The most significant electronic transitions at lower energy; b. ref [1]; c. TDA-B3LYP-D3/6-31G(d,p) with the exception of Mn and Br, treated with LANL2DZ pseudopotential and its valence; d. TDA-B3LYP-D3/6-31G(d,p). | | | | |

**Table S2**. Comparison between crystallographic and optimized structure of Mn(I) at B3LYP-D3/6-31G(d,p) level in the three environments considered

|  |  | | | | | | |
| --- | --- | --- | --- | --- | --- | --- | --- |
| **Distance (Å)** | X-ray_a_ | Mn1_W_ | Mn1_D_ | Mn1_T_ | Δ_W_ | Δ_D_ | Δ_T_ |
| Mn-N1 | 2.060 | 2.068 | 2.068 | 2.068 | 0.008 | 0.008 | 0.008 |
| Mn-N2 | 2.078 | 2.118 | 2.118 | 2.120 | 0.040 | 0.039 | 0.042 |
| Mn-Br | 2.539 | 2.549 | 2.548 | 2.540 | 0.009 | 0.008 | 0.001 |
| Mn-C1 | 1.802 | 1.786 | 1.786 | 1.785 | -0.016 | -0.016 | -0.017 |
| Mn-C2 | 1.801 | 1.804 | 1.804 | 1.804 | 0.002 | 0.002 | 0.003 |
| Mn-C3 | 1.820 | 1.796 | 1.796 | 1.797 | -0.025 | -0.025 | -0.023 |
| **Angle (degrees)** |  |  |  |  |  |  |  |
| N1-Mn-N2 | 80.4 | 80.4 | 80.4 | 80.3 | 0.0 | 0.0 | -0.1 |
| N2-Mn-C2 | 94.9 | 92.3 | 92.3 | 92.4 | -2.6 | -2.6 | -2.5 |
| C2-Mn-C3 | 89.8 | 91.5 | 91.5 | 91.5 | 1.7 | 1.7 | 1.7 |
| C3-Mn-N1 | 94.8 | 95.3 | 95.3 | 95.3 | 0.5 | 0.5 | 0.5 |
| C1-Mn-N1 | 93.4 | 91.3 | 91.3 | 91.3 | -2.1 | -2.1 | -2.1 |
| C1-Mn-N2 | 93.1 | 93.2 | 93.3 | 93.3 | 0.1 | 0.2 | 0.2 |
| C1-Mn-C2 | 89.2 | 93 | 93.1 | 93.1 | 3.8 | 3.9 | 3.9 |
| C1-Mn-C3 | 90.2 | 92.9 | 92.9 | 93.1 | 2.7 | 2.7 | 2.9 |
| Br-Mn-N1 | 87.5 | 86 | 80.0 | 85.9 | -1.5 | -7.5 | -1.6 |
| Br-Mn-N2 | 84.9 | 84.2 | 84.1 | 83.7 | -0.7 | -0.8 | -1.2 |
| Br-Mn-C2 | 89.7 | 89.4 | 89.4 | 89.4 | -0.3 | -0.3 | -0.3 |
| Br-Mn-C3 | 91.9 | 89.5 | 89.5 | 89.7 | -2.4 | -2.4 | -2.2 |
| Br-Mn-C1 | 177.6 | 176.5 | 176.5 | 176.3 | -1.1 | -1.1 | -1.3 |
| a. ref. [2] | | | | | | | |

**Table S3:** Computed NBO analysis for the reference complexes [Mn(CO)_6_]^+^, [Mn(CO)_4_(AQ)]^+^, MnBr(CO)_5_.

| **Complex** | **Donor (i)** | **Acceptor (j)** | ***E*^(2)^** | ***E*^(2)^_TOT_^a^** | ***ε_j_ – ε_i_*** | ***F*_(i,j)_** |
| --- | --- | --- | --- | --- | --- | --- |
| [Mn(CO)_6_]^+^ | n_(1)_Mn | π*_(1)_CO | 5.5 | 13.15 | 0.23 | 0.035 |
|  | n_(2)_Mn | π*_(1)_CO | 1.22 |  | 0.23 | 0.017 |
|  | n_(3)_Mn | π*_(1)_CO | 6.43 |  | 0.23 | 0.038 |
| [Mn(CO)_4_(AQ)]^+^   | n_(1)_Mn | π*_(1)_CO1 | 1.54 | 28.1 | 0.24 | 0.019 |
|  | n_(2)_Mn | π*_(1)_CO1 | 3.68 |  | 0.13 | 0.023 |
|  | n_(3)_Mn | π*_(1)_CO1 | 22.88 |  | 0.1 | 0.051 |
|  | n_(1)_Mn | π*_(1)_CO2 | 13.11 | 90.55 | 0.21 | 0.051 |
|  | n_(3)_Mn | π*_(1)_CO2 | 77.44 |  | 0.07 | 0.077 |
|  | n_(1)_Mn | π*_(1)_CO3 | 9.95 | 102.55 | 0.21 | 0.044 |
|  | n_(3)_Mn | π*_(1)_CO3 | 92.6 |  | 0.07 | 0.084 |
|  | n_(1)_Mn | π*_(1)_CO4 | 1.43 | 27.85 | 0.24 | 0.018 |
|  | n_(2)_Mn | π*_(1)_CO4 | 3.79 |  | 0.13 | 0.023 |
|  | n_(3)_Mn | π*_(1)_CO4 | 22.63 |  | 0.1 | 0.051 |
| MnBr(CO)_5_   | n_(2)_Mn | π*_(1)_CO1 | 0.54 | 88.21 | 0.11 | 0.008 |
|  | n_(3)_Mn | π*_(1)_CO1 | 87.67 |  | 0.11 | 0.098 |
|  | n_(1)_Mn | π*_(1)_CO2 | 15.17 | 24.71 | 0.21 | 0.057 |
|  | n_(3)_Mn | π*_(1)_CO2 | 9.54 |  | 0.11 | 0.032 |
|  | n_(1)_Mn | π*_(1)_CO3 | 15.13 | 24.71 | 0.21 | 0.057 |
|  | n_(2)_Mn | π*_(1)_CO3 | 9.58 |  | 0.11 | 0.032 |
|  | n_(1)_Mn | π*_(1)_CO4 | 15.22 | 24.6 | 0.21 | 0.057 |
|  | n_(3)_Mn | π*_(1)_CO4 | 9.38 |  | 0.11 | 0.032 |
|  | n_(1)_Mn | π*_(1)_CO5 | 15.25 | 24.5 | 0.21 | 0.057 |
|  | n_(2)_Mn | π*_(1)_CO5 | 9.25 |  | 0.11 | 0.032 |
| a. sum of π-backdonation to the same acceptor from different metal lone-pairs. | | | | | | |

**Table S4.** TDDFT outcomes of Mn1 complex in water, DMSO and THF

| **Complex** | **Band** | **ΔE (eV)** | **λ (nm)** | ***f*** | **MO contribution^a^** |
| --- | --- | --- | --- | --- | --- |
| **Mn1_W_** | I | 3.10 | 400 | 0.018 | HOMO → LUMO 49%;  H-1 → L 21% |
|  |  | 3.16 | 393 | 0.038 | H-1 → L 49%; H→ L 27% |
|  | II | 4.08 | 304 | 0.098 | H-3 → L 71%; H-5 → L 23% |
|  |  | 4.39 | 282 | 0.041 | H-5 → L 70% |
|  |  | 5.35 | 232 | 0.024 | H-5 → L 51%; H-3 → L+1 22% |
|  | III | 5.88 | 211 | 0.246 | H-6 → L+2 43% |
| **Mn1_D_** | I | 3.10 | 401 | 0.021 | HOMO → LUMO 53%;  H-1 → L 20% |
|  |  | 3.15 | 394 | 0.038 | H-1 → L 52%;  HOMO → LUMO 25% |
|  | II | 4.07 | 305 | 0.102 | H-3 → L 73%; H-5 → L 22% |
|  |  | 4.39 | 282 | 0.043 | H-5 → L 71% |
|  | III | 5.34 | 232 | 0.027 | H-5 → L+1 46%; H-3 → L+1 22% |
|  |  | 5.88 | 211 | 0.318 | H-6 → L+2 34% |
| **Mn1_T_** | I | 3.01 | 411 | 0.028 | HOMO → LUMO 77% |
|  |  | 3.06 | 405 | 0.023 | H-1 → L 73% |
|  | II | 3.96 | 313 | 0.096 | H-3 → L 80%; H-5 → L 16% |
|  |  | 4.39 | 283 | 0.059 | H-5 → L 77% |
|  | III | 5.85 | 212 | 0.112 | H-3 → L+5 42% |
|  |  | 5.93 | 209 | 0.124 | H-7 → L+1 17% |
| a. only contribution greater than 20% were considered. | | | | | |

**References**

[1] D. A. Habashy, R. M. Khaled, A. Y. Ahmed, K. Radacki, S. K. Ahmed, E. K. Tharwat, H. Magdy, A. Zeinhom, A. M. Mansour, Dalton Transactions, 2022, 51, 14041–14048.

[2] R. M. Khaled, A. Friedrich, M. A. Ragheb, N. T. Abdel-Ghani, A. M. Mansour, Dalton Transactions, 2020, 49, 9294–9305.
